# The emergence of inequality in social groups: Network structure and institutions affect the distribution of earnings in cooperation games

**DOI:** 10.1371/journal.pone.0200965

**Published:** 2018-07-20

**Authors:** Milena Tsvetkova, Claudia Wagner, Andrew Mao

**Affiliations:** 1 Department of Methodology, London School of Economics and Political Science, London, United Kingdom; 2 GESIS – Leibniz Institute for Social Sciences, Cologne, Germany; 3 Department of Management, Aarhus University, Aarhus, Denmark; Centre National de la Recherche Scientifique, FRANCE

## Abstract

From small communities to entire nations and society at large, inequality in wealth, social status, and power is one of the most pervasive and tenacious features of the social world. What causes inequality to emerge and persist? In this study, we investigate how the structure and rules of our interactions can increase inequality in social groups. Specifically, we look into the effects of four structural conditions—network structure, network fluidity, reputation tracking, and punishment institutions—on the distribution of earnings in network cooperation games. We analyze 33 experiments comprising 96 experimental conditions altogether. We find that there is more inequality in clustered networks compared to random networks, in fixed networks compared to randomly rewired and strategically updated networks, and in groups with punishment institutions compared to groups without. Secondary analyses suggest that the reasons inequality emerges under these conditions may have to do with the fact that fixed networks allow exploitation of the poor by the wealthy and clustered networks foster segregation between the poor and the wealthy, while the burden of costly punishment falls onto the poor, leaving them poorer. Surprisingly, we do not find evidence that inequality is affected by reputation in a systematic way but this could be because reputation needs to play out in a particular network environment in order to have an effect. Overall, our findings suggest possible strategies and interventions to decrease inequality and mitigate its negative impact, particularly in the context of mid- and large-sized organizations and online communities.

## Introduction

From social status hierarchies in kindergarten play groups and college fraternities [1] to pay dispersion at the workplace [2] and from extreme levels of popularity in online communities [3] to shockingly uneven distributions of material wealth within states [4], inequality takes many forms and occurs at many different levels of social organization. Why do we observe extreme distributions of outcomes when people only moderately vary in intelligence, physical abilities, and psychological traits? Why does inequality exist when people actually have strong preference for equity and fairness [5, 6]?

Evidently, we cannot explain inequality with human heterogeneity or preferences. Instead, social scientists have looked at social structure and its effect on behavior to explain the outcomes of individuals and groups. For example, researchers focusing on historical approaches have argued that certain global, state-level, and group-level processes and trends have disadvantaged some classes of individuals in terms of economic capital and political representation while enriching others [7–9]. Sociologists working on social stratification and mobility have observed that structural, network, and psychological factors, such as institutional arrangements, neighborhood and school characteristics, parents’ socioeconomic standing, peer influences, and innate ability and motivation, translate into individual outcomes such as social class, education, health, and income [10–12]. Social psychologists and behavioral economists have used controlled experiments to demonstrate that social differentiation and scarcity of resources affects individual behavior in a way that reinforces and enhances pre-existing differences [13–16].

While this research has greatly expanded our understanding of the extent, trends, and consequences of inequality in society, it does not paint a complete picture. Historical approaches focus on the macro-level and often ignore individuals, stratification research focuses on individuals but disregards interpersonal interactions, while behavioral research centers on small-group interactions but rarely compares groups on a macro-level. In many social settings, large groups of individuals interact in a way that their behavior and interactions drive group-level outcomes such as inequality, while the emerging group-level patterns in turn affect individual decision-making and actions. In such complex systems, the coevolution of interaction structure and individual behavior might create self-reinforcing feedback loops that amplify or exaggerate small initial and/or accidental differences into extreme distributions of outcomes [17]. Inequality, then, can be viewed as an emergent phenomenon that can play out differently under different structural and institutional constraints. This will be the case in mid- and large-sized social groups based on face-to-face or online interactions such as hunter-gatherer groups, agrarian villages, large work offices, schools, social media sites, online forums, and user-generated content communities.

Some research on how inequality emerges in complex social systems already exists. Theoretical work from within this paradigm has shown, for example, that population growth and preferential attachment [18], multiplicative gains and losses [19], and trade and inheritance [20] create and maintain inequality. Empirical research on this topic is more challenging since one needs to observe and manipulate multiple large social groups, which could be both costly and logistically complicated. Nevertheless, empirical work has also started to accumulate. From large-scale online experiments on cultural and attention markets, we know that due to social influence, the visibility of success increases inequality [21], even if the initial success was arbitrarily awarded [22]. In addition, experiments on cooperation games have shown that the visibility of wealth affects the cooperative behavior of the wealthy and exacerbates pre-existing inequality [23], reputation systems increase inequality by facilitating increasing returns from early cooperation [24], and inequality is reduced in networks where the very wealthy and the very poor are connected [25].

Extending this line of research, we present empirical evidence on how four structural conditions and institutional constraints affect the emergence of inequality in social groups. We investigate the effects of network structure, network fluidity, visibility of reputation, and punishment institutions. These conditions determine whether people interact within local clusters or with random others, whether they interact with the same others again and again, whether they have third-party information about others’ past behavior, and whether they can punish, at a cost, those who do not behave nicely. These are conditions that characterize some existing social groups more than others (compare, for example, the stable interactions in agrarian villages versus the constant recombination of nomadic groups) and can be implemented in the design of new formal organizations and online communities (for example, deciding whether to institute a reputation system in a local community classifieds website).

To gather observations on social groups, we use data from a large number of experiments on cooperation games in networks [26–43]. In the past couple of decades, these kinds of experiments have been most commonly used to study the emergence of cooperation and network formation. The experiments use games such as the Prisoner’s Dilemma and the Public Good Game in which participants have a choice between cooperating/contributing and defecting/doing nothing. Cooperation is costly and hence not individually rational but it has positive returns for the recipients so hence, it is collectively beneficial. Thus, the decision situation poses a social dilemma. Participants play the game repeatedly with numerous partners simultaneously. Further, different groups play in different network structures, with different information, or with additional action possibilities. This interaction setup models well situations in which members of large groups interact in subgroups and work together towards a common goal, such as rendering a service, producing a product, or increasing knowledge.

To study inequality, we look at the distribution of the points individuals accrue by the end of the game. These points can be understood as the individuals’ wealth, whether material or immaterial. For example, if we perceive cooperation as exchanging valuable information or useful advice, then the points will measure wealth in knowledge or wisdom.

The experiments we analyze were originally used to study the emergence of cooperation. The researchers found that network clustering, network stability, strategic network rewiring, and the possibility for reputation tracking and punishment increase the average level of cooperation. With the exception of costly punishment which reduces point earnings, more cooperation implies higher average number of points, i.e. higher wealth. However, how do the different structural factors affect the distribution of wealth in the interaction groups? This question has not been systematically investigated before. The answer is nontrivial as high levels of cooperation can have opposite effects on inequality, depending on how cooperators and defectors are clustered and interconnected. Further, some structural conditions may affect the behavior of different individuals differently, either erasing or exacerbating emerging wealth disparities.

### Hypotheses

In what follows, we discuss how network structure, network fluidity, reputation tracking, and punishment institutions affect the network composition and the behavior of individuals in group cooperation games and hypothesize what this implies for inequality. In the Results section, we use the experimental data to test the hypotheses causally at the group level and to provide some insights as to the actual behavioral and interaction mechanisms at play.

#### Network structure

From previous research we know that networks with high levels of clustering, whereby an individual’s neighbors tend to be neighbors with each other, are expected to foster forward-looking behavior, imitation, and social reinforcement and promote the emergence of cooperative clusters [30, 38]. This implies that cooperators and defectors are more likely to become segregated in their own separate clusters. Since cooperators interacting with cooperators benefit, while defectors interacting with defectors lose out, we would expect higher inequality in clustered networks compared to networks that are more random and without local structure. To relate this to a realistic setting, this hypothesis would imply, for example, that schools with stringent degree requirements that concentrate most of learning within classrooms or majors will be marked with higher inequality in educational outcomes than schools that allow elective courses and more uniform interactions among students, assuming that student cooperation and collaboration enhance learning.

#### Network fluidity

The above rationale assumed that the networks are relatively stable and individuals interact repeatedly with the same partners. If the partnerships are more volatile, cooperative clusters are less likely to emerge unless individuals self-select into them. Moreover, fixed networks enable exploitation: knowing that your cooperating partners cannot leave, you may as well start free-riding on their efforts and contributions; and since defectors gain disproportionately more when they exploit cooperators, inequality may increase. In contrast, exploitative behavior is not possible in networks that are regularly reshuffled and can be costlessly punished with exclusion in networks that allow choosing partners strategically. In short, higher clustering of cooperators and more exploitation by defectors would imply higher inequality in fixed networks compared to networks in which interaction partners change randomly. But the comparison between fixed networks and networks in which individuals can select their interaction partners is less straightforward. The latter structures preclude exploitative defectors due to the possibility to exclude them but enable even higher segregation due to the possibility to self-select into cooperative clusters. However, previous research has clearly shown that strategic partner selection essentially eradicates defection [40, 44, 45], so again, we would expect fixed networks to result in more inequality. To extrapolate to a concrete social setting, the hypotheses would imply, for example, that sedentary agrarian villages, which are marked by relatively stable social relations, have higher inequality in material and immaterial wealth than nomadic groups, which tend to change composition and structure on a regular basis.

#### Reputation tracking

Reputation institutions, which make available information about everyone’s past behavior towards others, present another factor that is expected to increase inequality in social groups. Previous research has shown that individuals are more likely to select partners who have reputation as cooperators [24], as well as more likely to cooperate with them [29]. This implies that initial differences in cooperative behavior could have long-lasting effects that get reinforced and exaggerated over time. As a result, social groups where reputation is visible will have higher inequality than groups without reputation tracking. Such an effect would have important implications for the design of online marketplaces and social media communities, for example.

#### Punishment institutions

Finally, the possibility for peer punishment is also expected to increase inequality in social groups. Previous research on the Prisoner’s Dilemma game has shown that the poor are more likely to pay to punish defecting partners; in contrast, the wealthy are more likely to use a tit-for-tat strategy and defect in response to defection [32]. This suggests the poor will use costly punishment disproportionately, which will make them even poorer compared to the wealthy. As a result, punishment will increase not only cooperation, but also inequality. To give a concrete example, this hypothesis would imply that communities relying on peer monitoring and punishment would have higher inequality than communities in which everyone is required to contribute equally, or even proportionally to wealth, to a single centralized sanctioning institution.

## Results

In the following, we investigate whether inequality is higher in groups interacting in clustered networks compared to random networks, in fixed networks compared to randomly or strategically rewired networks, with punishment institutions compared to no possibility for costly punishment, and with reputation tracking compared to without. In addition, we look for possible explanations for the observed results related to the clustering of cooperators, ease of excluding exploitative defectors, and differential punishment behavior between successful and unsuccessful players.

Detailed information about the data, the definitions, the measures and the statistical tests we use in the study can be found at the end of the article in the section Materials and Methods. Table 1 summarizes the data we use for the analyses. The data come from 18 previously published studies and to identify the different data sets, we use an abbreviation constructed from the first four letters of the first author’s last name and the last two digits of the study’s year of publication. Some of the studies have substantially different orthogonal treatment that allows us to split them into separate experiments, which we mark with a lower-case letter appended at the end of the name. This gives us 33 experiments. The experiments vary in terms of the game used (Prisoner’s Dilemma, Public Good, or helping), the size of the interaction group (from 8 to 625), the number of interaction groups (from 3 to 84), the number of players’ interaction partners (from 1 to all other group members), the number of periods of interaction (from 6 to more than 90), and whether the experimental design is within-subject (participants play in more than one experimental group) or within-group (groups play in more than one experimental condition). Further, each experiment can have multiple treatment conditions.

**Table 1:** Description of the experiments used in the analyses.

Exp	Game	*N*_*g*_	*Size*_*g*_	*N*_*i*_	*k*	*T*	Network	Fluid	Reput	Punish
**Network Structure**										
CASS07	PD: 4*p*_*C*_, 0, 5*p*_*C*_, *p*_*D*_	11	18	72	≈4	78–84	ran,sw,cyc	fix	>1	no
KIRC07a	PD: 5*C*, 0, 4 + 5*C*, 4	11	8–20	168	4	80	cyc,cliq	fix	0	no
KIRC07b	PD: 5*C*, 0, 4 + 5*C*, 4	11	8–20	190	4	80	cyc,cliq	fix	1	no
SURI11	PG: 10 − *c*_*i*_ + 0.4〈*c*_*j*_〉	30	24	109	5	10	ran,sw,cyc,pcliq,cliq	fix	1	no
WANG12	PD: 4*C*, *aD*, 7*C*, *bD*	10	24	108	5	12	ran,cliq	fix	5	no
**Network Fluidity**										
GRAC12a	PD: 7*C*, 0, 10*C*, 0	2/1	625	625	4	51	lat	shuf,fix	1	no
GRAC12b	PD: 7*C*, 0, 10*C*, 0	2/1	604	604	2–16	58	sf	shuf,fix	1	no
GRUJ10	PD: 7*C*, 0, 10*C*, 0	3/1	169	169	8	47–60	lat	shuf,fix	1	no
RAND14a	PD: 40*C* − 20, −20, 40*C*, 0	8	15–34	210	2	15	cyc	shuf,fix	1	no
RAND14b	PD: 40*C* − 20, −20, 40*C*, 0	8	15–34	193	2	15	cyc	shuf,fix	1	no
RAND14c	PD: 60*C* − 40, −40, 40*C*, 0	8	15–34	210	4	15	cyc	shuf,fix	1	no
TRAU10	PD: 0.3*C*, 0, 0.4*C*, 0.1*D*	25	16	400	4	25	lat	shuf,fix	1	no
PAGE05a	PG: 10 − *c*_*i*_ + 0.4 ∑ *c*_*j*_	8	16	128	3	20	cliq	fix,strat	avg	no
PAGE05b	PG: 10 − *c*_*i*_ + 0.4 ∑ *c*_*j*_	8	16	128	3	20	cliq	fix,strat	avg	yes
WANG12a	PD: 4*C*, *aD*, 7*C*, *bD*	43	24	108	(5)	12	(ran)	fix,strat	5	no
WANG12b	PD: 4*C*, *aD*, 7*C*, *bD*	41	24	108	(5)	12	(cliq)	fix,strat	5	no
**Reputation Tracking**										
BOLT05a	HG: −0.25, 1.25	6	16	96	1	14	pair	shuf	0,1,1+1	no
BOLT05b	HG: −0.75, 1.25	6	16	96	1	14	pair	shuf	0,1,1+1	no
CUES15	PD: 7*C*, 0, 10*C*, 0	22/11	17–25	243	(4)	25	cyc	strat	0,1,3,5	no
KAME17a	PG: 10 − *c*_*i*_ + 0.65 ∑ *c*_*j*_	12	10	120	1	40	pair	strat	0,50%,100%	no
KAME17b	PG: 10 − *c*_*i*_ + 0.85 ∑ *c*_*j*_	13	10	130	1	40	pair	strat	0,50%,100%	no
KIRC07a	PD: 5*C*, 0, 4 + 5*C*, 4	9	8–20	158	4	80	cyc	fix	0,1	no
KIRC07b	PD: 5*C*, 0, 4 + 5*C*, 4	6	8–20	95	4	80	cliq	fix	0,1	no
KIRC07c	PD: 5*C*, 0, 4 + 5*C*, 4	7	8–20	105	4	80	lcliq	fix	0,1	no
SEIN06	HG: −150, 250	8	14	112	1	>90	pair	shuf	1,6	no
**Punishment Institutions**										
CASA09	PG: 20 − *c*_*i*_ + 0.4 ∑ *c*_*j*_	24/12	20	240	4	10	cliq	shuf	0	no,yes,seq,cons
DREB08a	PD: 1, −2, 2, 0	2	≈26	58	1	71	pair	shuf	1	no,yes
DREB08b	PD: 1, −2, 4, 0	2	≈26	46	1	87	pair	shuf	1	no,yes
FEHR02	PG: 20 − *c*_*i*_ + 0.4 ∑ *c*_*j*_	20/10	24	236	3	6	cliq	shuf	0	no,yes
NIKI08	PG: 20 − *c*_*i*_ + 0.4 ∑ *c*_*j*_	12/8	12	96	3	10	cliq	shuf	1	no,yes
OGOR09	PG: 20 − *c*_*i*_ + 0.5 ∑ *c*_*j*_	10/6	20–24	136	3	6	cliq	shuf	0	no,yes,solo
PAGE05a	PG: 10 − *c*_*i*_ + 0.4 ∑ *c*_*j*_	8	16	128	4	20	cliq	fix	avg	no,yes
PAGE05b	PG: 10 − *c*_*i*_ + 0.4 ∑ *c*_*j*_	8	16	128	4	20	cliq	strat	avg	no,yes

The games played in the experiments are Prisoner’s Dilemma (PD), Public Good game (PG), and helping game (HG); payoffs are shown for CC, CD, DC, DD for Prisoner’s Dilemma and for C, D for helping game, where *p*_*C*_ (*p*_*D*_) and *C* (*D*) are, respectively, the proportion and the number of neighbors who cooperate (defect) and *c*_*i*_ (*c*_*j*_) is the amount contributed by the player (the player’s partners). *N*_*g*_ = number of experimental groups, with / separating the number of group observations from the number of unique groups for experiments with within-group design, *Size*_*g*_ = group size, *N*_*i*_ = number of unique participants, *k* = number of partners, *T* = number of periods. Network refers to network structure (ran = random, sw = small world, cyc = cycle, cliq = cliques, pcliq = paired cliques, lcliq = large cliques, lat = lattice, sf = scale free, pair = pairs). Fluid refers to network fluidity (fix = fixed, shuf = randomly rewired, strat = strategically rewired). Reput refers to reputation tracking with the number (percentage) indicating the number (percentage) of partner’s past actions that is observed and avg indicating that only average behavior is observed; 1 + 1 refers to observing partner’s action in last period, as well as the action of the partner’s partner from the period before that. Punish refers to punishment institutions (no = no punishment, yes = players can punish partners, seq = players punish partners sequentially, cons = a player is punished if at least two partners agree to punish them, solo = one player is randomly selected each period to punish).

Since we rely on experimental data, our tests of the main hypotheses are causal. However, since we execute our analyses at the group level, we cannot clearly disentangle the individual-level mechanisms. Nevertheless, we use observational analysis techniques to provide some descriptive and exploratory evidence as to the extent to which the assumed mechanisms play a role.

### Inequality

We first compare the level of inequality at the end of the games between control-treatment pairs. We measure inequality with the Gini coefficient and use the Mann-Whitney test to assess differences between the control and each treatment. However, this evidence is not always sufficient because the experiments were not designed to test group-level hypotheses nor to study inequality, so only on several occasions we have large enough effect sizes and enough statistical power to provide evidence at the level of a single experiment. Instead, what we do is to treat each of the very different control-treatment pairs as independent trials and to focus on the direction of the effect, rather than its size and significance. This allows us to use an established meta-analytic approach known as the sign test [46]. In essence, the sign test establishes whether the distribution of observed effects is significantly different from the 50 negative/50 positive distribution we expect if, in reality, there was no causal effect from the particular structural condition on inequality (see Materials and methods).

#### Network structure

**Fig 1:**
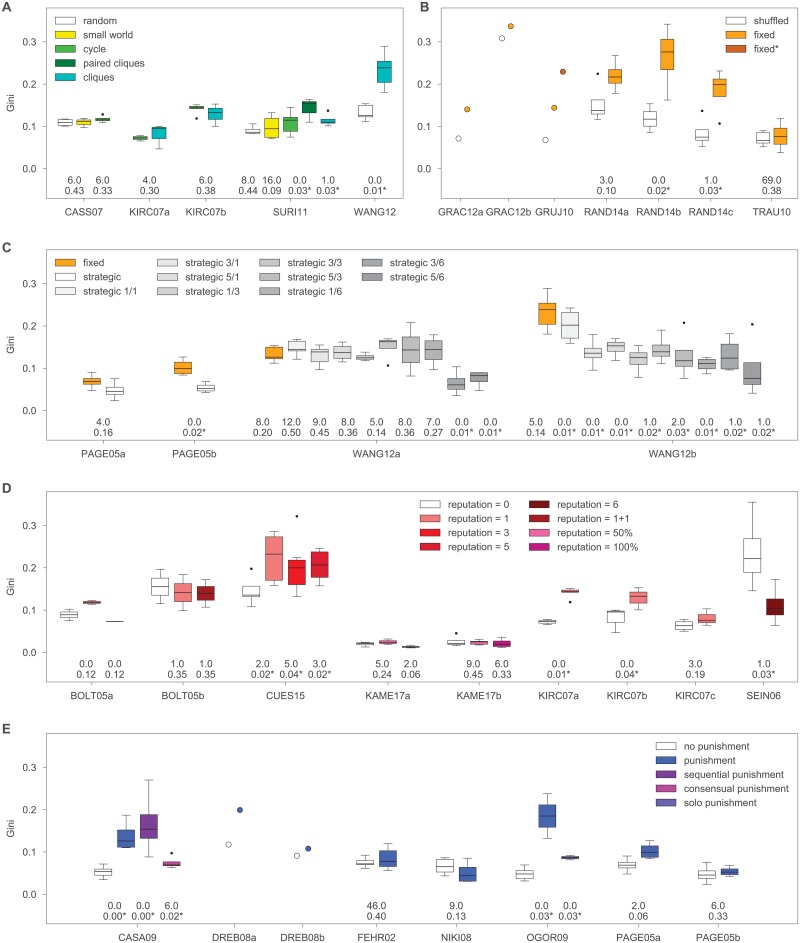
The effects on inequality from (A) network structure, (B, C) network fluidity, (D) reputation tracking, and (E) punishment institutions. The figure shows boxplots for each experimental condition and results from the Mann-Whitney tests comparing each treatment condition to the control condition within each experiment (Mann-Whitney *U* on top and *p*-value on bottom, with asterisk if *p* < 0.05). For each experiment, the first bar shown in the figure is the control condition and each test result compares this control condition to the treatment conditions represented by each next bar in order. Explanation of the experimental conditions can be found in Table 1. In addition, for GRUJ10, “fixed*” refers to a second fixed condition played by the same group and for WANG12, “strategic *a*/*b*” means that participants can make up to *a* partner updates in each of *b* partner-update periods of the game.

For the effect of network structure on inequality, we compare the Gini coefficient in the least clustered networks (usually the random networks) to the other types of networks in the experiment. In SURI11 we find that paired cliques and cliques have significantly higher inequality than random networks (Fig 1A). We confirm the finding about clique networks in WANG12, which uses the Prisoner’s Dilemma instead of the Public Goods game. The results for the other types of networks and in the other experiments are not statistically significant but overall, eight of the nine control-treatment differences point in the same direction. This indicates that more clustered networks have significantly higher inequality (Binomial test, 1-sided *p* = 0.020).

#### Network fluidity

For the effect of network fluidity, we compare the Gini coefficients in fixed networks to randomly rewired, or shuffled, networks (Fig 1B) and to strategically updated networks (Fig 1C). As expected, fixed networks have higher inequality than shuffled networks (Binomial test, 1-sided *p* = 0.008). The direction of the effect is the same in all seven experiments, although it is significant only in two of the RAND14 setups. Fixed networks also appear to have higher inequality than strategically updated networks. The results are significant in the PAGE05 experiment with punishment and in the WANG12 experiments starting from clique networks and varying the level of rewiring. These results were also found in another published study that investigated the effect of strategic network updating on inequality [47]. Based on the data we analyze here, 14 out of the 20 control-treatment differences show higher inequality for fixed networks compared to networks with strategic updating. The sign test is marginally not significant at the 0.05 level (Binomial test, 1-sided *p* = 0.058). However, adding the results from [47], for which we did not have raw data, gives us 22 out of 28 and stronger statistical evidence overall (Binomial test, 1-sided *p* = 0.002).

#### Reputation tracking

For the effect of reputation, we compare any conditions with visible reputation to the condition without. We expected that reputation tracking increases inequality but the effects in the different experiments appear to go in both directions (Fig 1D). We observe significantly higher inequality in groups with reputation compared to without in CUES15 and KIRC07 but exactly the opposite result in SEIN06, while in BOLT05 and KAME17 the effects are in both directions and non-significant. Overall, only nine out of the 15 differences point in the expected direction (Binomial test, 1-sided *p* = 0.304). This suggests that either there is no effect of reputation, or the effect of reputation is complex and crucially depends on some of the other structural conditions.

#### Punishment institutions

Finally, groups with punishment have higher levels of inequality in ten out of the 11 control-treatment comparisons with the effects being significant in the CASA09 and OGOR09 experiments (Fig 1D). In other words, the existence of punishment institutions significantly increases inequality (Binomial test, 1-sided *p* = 0.006).

### Mechanisms

We next analyze the individual behavior and the network dynamics in the experiments to investigate possible explanations for the observed higher levels of inequality. Unfortunately, we do not have individual and network data for all of the experiments, so we conduct our analyses on a smaller subset. We use observational data techniques to provide suggestive evidence on the different behavioral mechanisms behind the main effects that we presupposed.

First, we investigate the extent to which the emergence of cooperative clusters could explain the higher inequality we observe in fixed and clustered networks. We measure the clustering of cooperators with the network assortativity by cooperativeness in the final period. We find that assortativity by cooperativeness is significantly higher than expected for the most clustered fixed networks in SURI11 and CASS07 (S1A Fig), which also show higher inequality than random networks (Fig 1A). Furthermore, in SURI11 we also find that cycles and paired cliques have significantly higher assortativity by cooperativeness than random networks. Although these effects do not explain all of the results, they suggest that the emergence of cooperative clusters is a plausible pathway via which clustered networks increase inequality, at least in some cases.

Exploitation, whereby individuals get tempted to defect in fixed networks since the cooperators around them cannot move away, could be another pathway for inequality to increase. We find suggestive evidence for this in S1B and S2B Figs, which show that in many cases fixed networks have lower network assortativity by cooperativeness and wealth than randomly rewired or strategic networks. Additionally, we see that in fixed networks both defectors and cooperators could achieve the highest wealth (S3–S5 Figs). This suggests that, when networks are fixed, inequality could increase due to both the clustering of the cooperators and the exploitation of these clusters by defectors.

To investigate whether punishment institutions increase inequality because the poor pay for punishment disproportionately more than the wealthy, we correlate the tendency to use punishment with wealth and cooperativeness. In alignment with [32], we find overwhelming evidence that this is the case: in all 11 punishment treatments, wealth and the use of punishment are negatively correlated (S6 Fig; Binomial test, 1-sided *p* = 0.000). This finding cannot be explained with cooperativeness, as we actually find no systematic relation between being cooperative and using punishment (S7 Fig; Binomial test, 1-sided *p* = 0.274). Of course, with the data we have we cannot establish whether the wealthy do not punish or whether those who do not punish become wealthy.

## Discussion

We analyzed data from a number of experiments on repeated cooperation games in networks to study how network structure, network fluidity, reputation tracking, and punishment institutions affect inequality in social groups. The results confirm that there is more inequality in clustered networks compared to random networks, fixed networks compared to randomly rewired networks, and groups with punishment institutions compared to groups without. We also find convincing evidence that fixed networks have higher inequality than networks with strategic updating. These conditions give rise to inequality because they affect either the structure of interactions or the behavior of individuals in a way that differences between the wealthy and the poor grow. Fixed networks allow exploitation of the poor by the wealthy (similarly to the dynamic discovered in [23]), clustered fixed networks foster segregation between the poor and the wealthy, and costly punishment imposes a disproportionate burden on the poor, making them poorer.

Surprisingly, we do not find evidence that inequality increases when reputation is visible. Given that the experiments we analyze differ on many dimensions, we are unwilling to take this as a proof that reputation has no effect on inequality; rather, we believe that reputation needs to interact with another structural condition to play a role. In the experiments that we find to confirm the expected effect, interaction is situated in a network (whether fixed or strategically updated) and not in pairs that get re-matched in each period. This would suggest that the effect of reputation is perhaps only enacted in more stable networks and perhaps even more so when exclusion is possible. Future research should investigate these ideas.

The findings from our study should be interpreted within the confines of our data-driven approach. The experiments we analyzed were not designed to study inequality and they were rarely large enough to test group-level hypotheses. However, although only a handful of the individual experiments may be convincing by themselves, the fact that we found the same effects across so many different experimental setups provides strong evidence for the hypothesized causes of inequality. Nevertheless, as is often the case with scientific research, our study is not the definitive answer to the question of what causes inequality to emerge in social groups. Ideally, our findings will be replicated and extended using carefully designed large-group experiments, as well as validated with observational data from different social contexts. Further, future research should develop experiments to test a more complete set of structural conditions and mechanisms that cause inequality. For example, it is worth mentioning that the levels of inequality we observe in our experiments are relatively low. Compared to national-level wealth inequality, the highest Gini coefficient in our data of 0.34 is well below what we observe in any country [4]. This is to be expected because the experiments we analyzed did not allow for the investment of wealth, as in [48]. Thus, one possible avenue for future research is to experimentally test how different investment rules and opportunities affect inequality in social groups.

The significance of our findings depends on the extent to which one considers inequality to be an undesirable social problem. One argument against worrying about inequality is that it might fairly reflect differences between individuals. In the context of network games, perhaps inequality is not so bad if cooperators are rewarded, while defectors struggle. Unfortunately, as we saw above, this is not always the case (S3–S5 Figs). Another argument for maintaining inequality is that inequality increases the collective wealth and well-being is thus essential for prosperity. Again, our data show poor support for this argument: there is no evidence for a systematic relationship between inequality and average wealth (S8 and S9 Figs; Binomial test, 1-sided *p* = 0.243). In other words, slightly different incentives, information, or rules may not affect the collective wealth but may significantly skew how it is distributed in the group, irrespective of common-sense ideas of fairness; inequality in social groups can indeed be problematic.

Overall, our study suggests that highly clustered fixed networks where punishment is possible have higher inequality than more unstable communities. This finding has an interesting parallel to previous research showing that sedentary agrarian communities have higher inequality than nomadic communities [49, 50]. This research suggested that inequality is higher in agrarian communities due to the possibility to accumulate wealth and transmit it to subsequent generations. Our study points to additional structural conditions that could explain the difference: agrarian communities are likely to have higher inequality because they are stable and with functioning punishment institutions. These structural characteristics enable self-reinforcing social processes that create and perpetuate divisions between the wealthy and the poor. Our findings also provide insights for managing inequality in organizations such as companies, universities, hospitals, and online communities around user-generated content sites and peer-to-peer markets. In such social settings, more dynamic and less locally clustered interaction structures that do not encourage costly mutual sanctioning could enable a more even distribution of resources and gains.

## Materials and methods

### Data

To identify suitable data, we conducted online searches on the Scopus database and the Google Scholar search engine. Our searches looked for articles with the words “cooperation”, “game”, and “experiment”, together with one of “punishment”, “reputation”, or “network”, in their title and abstract. When we identified a relevant study, we then also investigated the articles cited by it. In general, we restricted our interest to experiments in groups of at least 10 (although we did not exclude the smaller eight-person groups in [31] for our analyses). We thus identified 21 relevant studies and contacted the corresponding authors. In the end, we obtained data for 18 of them.

### Measures

We measure wealth with the amount of points the player has amassed at the end of the game. We then use the Gini coefficient to measure inequality in wealth. A Gini coefficient of 0 indicates perfect equality where all group members have equal resources, while 1 indicates perfect inequality where a single individual commands all of the resources. The Gini coefficient is the most common measure of inequality but nevertheless has some limitations. In particular, it is sensitive to changes in inequality among those in the middle of the distribution and not so much to those at the lower end of the distribution [51]. We replicated our analyses using another measure, the Theil index, and the results are qualitatively similar.

To measure cooperativeness, we calculate the mean number (or amount) of contributions over all periods. To measure the tendency for clustering, we calculate the network assortativity by cooperativeness and wealth in the final period. For continuous measures, which is the case for cooperativeness and wealth here, network assortativity is essentially the correlation in the measure for all connected pairs [52].

### Statistical tests

To test differences between experimental conditions for inequality, we use the Mann-Whitney U test. This is a non-parametric test that does not assume a normal distribution for the residuals. It essentially checks against the null hypothesis that a randomly selected value from one condition would be equally likely to be less than or greater than a randomly selected value from the other condition. For the experiments that have a within-group design, whereby groups interact in more than one experimental condition, we considered using the Wilcoxon signed rank sum test but unfortunately, the sample sizes were always too small to have reliable results. Consequently, we only report the Mann-Whitney U in Fig 1. This limitation is not severe because our main results are based on the meta-analysis of the direction of all control-treatment differences, rather than the precise test of any individual one.

To test the statistical significance of the observed differences between control and treatment across all relevant experiments, we use a meta-analysis technique known as the sign test [46]. This technique allows us to focus on whether the effect exists, rather than to accurately measure its size. We first count the number of positive and negative effects, regardless of whether they are statistically significant. We then conduct the Binomial test, testing against the null hypothesis that there is no effect in reality and thus negative and positive effects are equally likely to occur by chance. This approach is somewhat limited because it does not take into account the amount of evidence: neither the effect magnitudes nor the sample sizes. Yet, it is best suited for the research question and experimental data we have. First, although meta analyses typically aim to estimate the effect size, effect sizes in controlled social experiments are not very meaningful as they are highly sensitive to the experimental design and, in particular, aspects such as the framing of the decision situation, the monetary incentives, the experience of the participant pool, and the experimenter demand effect [53]. In relying on experimental data, our major objective here is to prove a causal relationship and the sign test is perfectly sufficient for that. Second, pooling effect sizes from studies with different designs and research populations is usually problematic in meta analysis but for us, the variation in our data sources is a boon, not a drawback. Experiments are often criticized for their lack of replicability and validity, so being able to confirm the same effect repeatedly in completely different experimental settings only reaffirms the robustness of the result.

To test the significance of the observed network assortativity by cooperativeness and wealth, we need a suitable baseline. The reason is that network assortativity depends both on the connectivity patterns and on the distribution of values of interest. For the baseline, we take the actual final structure in the networks, randomly shuffle the nodes’ cooperativeness and wealth values, and estimate the new assortativity. We repeat this 2000 times, which gives us an expected distribution against which we can estimate the z-score for the empirically observed value. The z-score is estimated using *Z* = (*X*_*obs*_ − *μ*(*X*_*ran*_))/*σ*(*X*_*ran*_), where *X*_*obs*_ is the assortativity in the observed network, *X*_*ran*_ is the network assortativity in the randomly shuffled network, *μ* is the mean, and *σ* is the standard deviation. If the z-score is above 1.96 (below −1.96), it implies that the chance to obtain the high (low) network assortativity we actually observe under the assumption that the network formation processes are random is less than 5%. Regardless of whether the network assortativity by cooperation and wealth is significantly different than what we would expect by chance, we can also test whether it is significantly different between experimental conditions. As with inequality, we use Mann-Whitney test to do this (S1 and S2 Figs).

To estimate the correlations between individual wealth and cooperativeness, wealth and the use of punishment, cooperativeness and the use of punishment, as well as group inequality and wealth (S3–S9 Figs), we use ordinary least-square regressions with standard errors accounting for clustering by experimental groups.

## Supporting information

S1 Fig Z-scores for network assortativity by cooperativeness for fixed and strategically rewired networks from a subset of the experiments on (A) network structure, (B) network fluidity, and (C) reputation tracking. The z-score shows to what extent the observed assortativity by cooperativeness for each experimental condition deviates from what we expect to see in a network with the same structure but with cooperativeness randomly assigned to nodes. The figure shows the z-score distribution and results from the Mann-Whitney test comparing each treatment condition to the control condition (Mann-Whitney *U* on top and *p*-value on bottom, with asterisk if *p* < 0.05). The grey band spans from *Z* = −1.96 to *Z* = 1.96, equivalent to *p* ≥ 0.05. For each experiment, the first bar shown in the figure is the control condition and each test result compares this control condition to the treatment conditions represented by each next bar in order.
(PDF)

S2 Fig Z-scores for network assortativity by wealth for fixed and strategically rewired networks from a subset of the experiments on (A) network structure, (B) network fluidity, and (C) reputation tracking. The z-score shows to what extent the observed assortativity by wealth for each experimental condition deviates from what we expect to see in a network with the same structure but with wealth randomly assigned to nodes. The figure shows the z-score distribution and results from the Mann-Whitney test comparing each treatment condition to the control condition (Mann-Whitney *U* on top and *p*-value on bottom, with asterisk if *p* < 0.05). The grey band spans from *Z* = −1.96 to *Z* = 1.96, equivalent to *p* ≥ 0.05. For each experiment, the first bar shown in the figure is the control condition and each test result compares this control condition to the treatment conditions represented by each next bar in order.
(PDF)

S3 Fig The relationship between individual cooperativeness and individual wealth in the conditions from the experiments on network structure. In each plot, values for individuals in the same interaction group are shown with the same symbol. The figure also shows fitted curves and estimates from ordinary least-square regressions (standardized regression coefficient for linear term on top and quadratic term on bottom, including *p*-values in brackets, with asterisk if *p* < 0.05). The standard errors in the regression models are estimated with correction for clustering by experimental group.
(PDF)

S4 Fig The relationship between individual cooperativeness and individual wealth in the conditions from the experiments on network fluidity. In each plot, values for individuals in the same interaction group are shown with the same symbol. The figure also shows fitted curves and estimates from ordinary least-square regressions (standardized regression coefficient for linear term on top and quadratic term on bottom, including *p*-values in brackets, with asterisk if *p* < 0.05). The standard errors in the regression models are estimated with correction for clustering by experimental group.
(PDF)

S5 Fig The relationship between individual cooperativeness and individual wealth in the conditions from the experiments on network fluidity. In each plot, values for individuals in the same interaction group are shown with the same symbol. The figure also shows fitted curves and estimates from ordinary least-square regressions (standardized regression coefficient for linear term on top and quadratic term on bottom, including *p*-values in brackets, with asterisk if *p* < 0.05). The standard errors in the regression models are estimated with correction for clustering by experimental group.
(PDF)

S6 Fig The relationship between individual use of punishment and individual wealth in the treatment conditions with punishment. In each plot, values for individuals in the same interaction group are shown with the same symbol. The figure also shows fitted lines and estimates from ordinary least-square regressions (standardized regression coefficient, equivalent to the Pearson correlation, on top and *p*-value on bottom, with asterisk if *p* < 0.05). The standard errors in the regression models are estimated with correction for clustering by experimental group.
(PDF)

S7 Fig The relationship between individual use of punishment and individual cooperativeness in the treatment conditions with punishment. In each plot, values for individuals in the same interaction group are shown with the same symbol. The figure also shows fitted lines and estimates from ordinary least-square regressions (standardized regression coefficient, equivalent to the Pearson correlation, on top and *p*-value on bottom, with asterisk if *p* < 0.05). The standard errors in the regression models are estimated with correction for clustering by experimental group.
(PDF)

S8 Fig The relationship between group average wealth and group inequality in the experiments on network structure and network fluidity. The figure shows fitted lines and estimates from ordinary least-square regressions (standardized regression coefficient, equivalent to the Pearson correlation, on top and *p*-value on bottom, with asterisk if *p* < 0.05). The standard errors in the regression models are estimated with correction for clustering by experimental group. Colors correspond to experimental conditions as in Fig 1A–1C.
(PDF)

S9 Fig The relationship between group average wealth and group inequality in the experiments on reputation and punishment. The figure shows fitted lines and estimates from ordinary least-square regressions (standardized regression coefficient, equivalent to the Pearson correlation, on top and *p*-value on bottom, with asterisk if *p* < 0.05). The standard errors in the regression models are estimated with correction for clustering by experimental group. Colors correspond to experimental conditions as in Fig 1D and 1E.
(PDF)
