# Supplementary figures and images for: The emergence of inequality in social groups: Network structure and institutions affect the distribution of earnings in cooperation games
Source: PLoS One. 2018 Jul 20;13(7):e0200965. doi: 10.1371/journal.pone.0200965 (PMC6054378; doi:10.1371/journal.pone.0200965)

**A**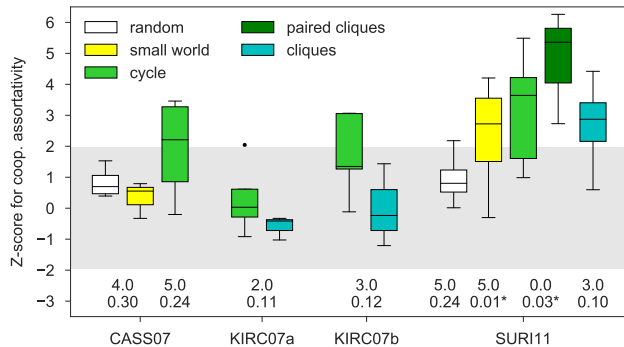**B**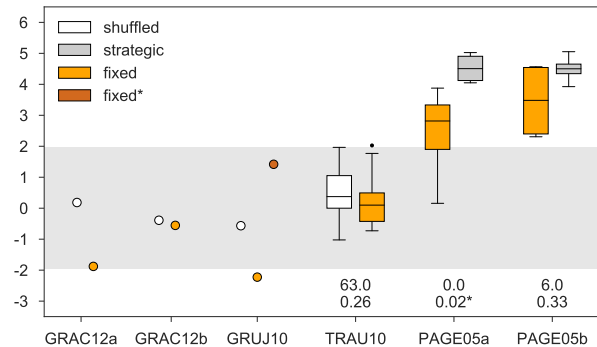**C**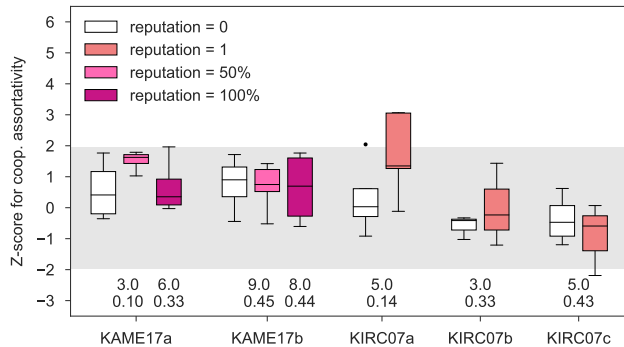

Supplement: S1 Fig — The z-score shows to what extent the observed assortativity by cooperativeness for each experimental condition deviates from what we expect to see in a network with the same structure but with cooperativeness randomly assigned to nodes. The figure shows the z-score distribution and results from the Mann-Whitney test comparing each treatment condition to the control condition (Mann-Whitney U on top and p-value on bottom, with asterisk if p < 0.05). The grey band spans from Z = −1.96 to Z = 1.96, equivalent to p ≥ 0.05. For each experiment, the first bar shown in the figure is the control condition and each test result compares this control condition to the treatment conditions represented by each next bar in order. (PDF) [file pone.0200965.s001.pdf]

**A**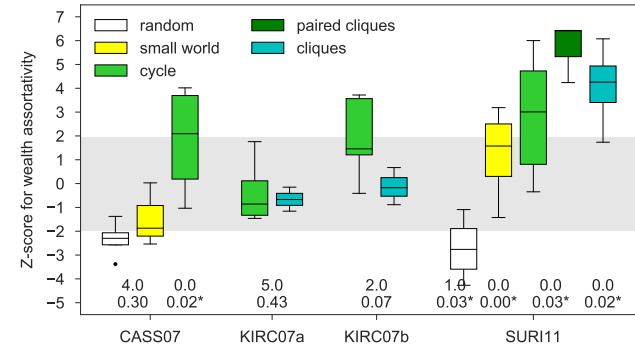**B**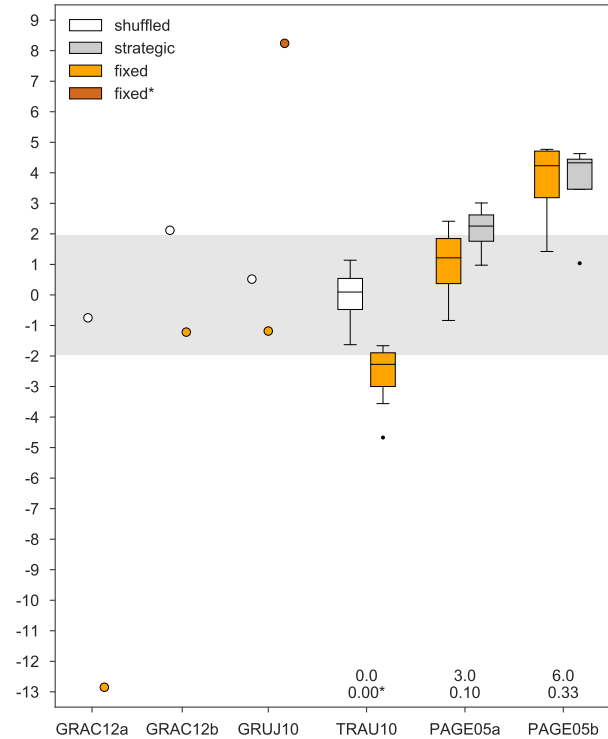**C**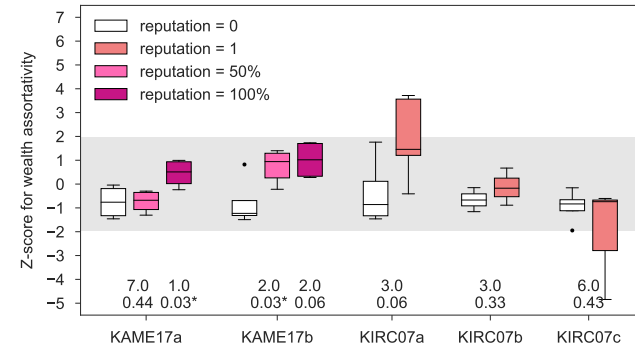

Supplement: S2 Fig — The z-score shows to what extent the observed assortativity by wealth for each experimental condition deviates from what we expect to see in a network with the same structure but with wealth randomly assigned to nodes. The figure shows the z-score distribution and results from the Mann-Whitney test comparing each treatment condition to the control condition (Mann-Whitney U on top and p-value on bottom, with asterisk if p < 0.05). The grey band spans from Z = −1.96 to Z = 1.96, equivalent to p ≥ 0.05. For each experiment, the first bar shown in the figure is the control condition and each test result compares this control condition to the treatment conditions represented by each next bar in order. (PDF) [file pone.0200965.s002.pdf]

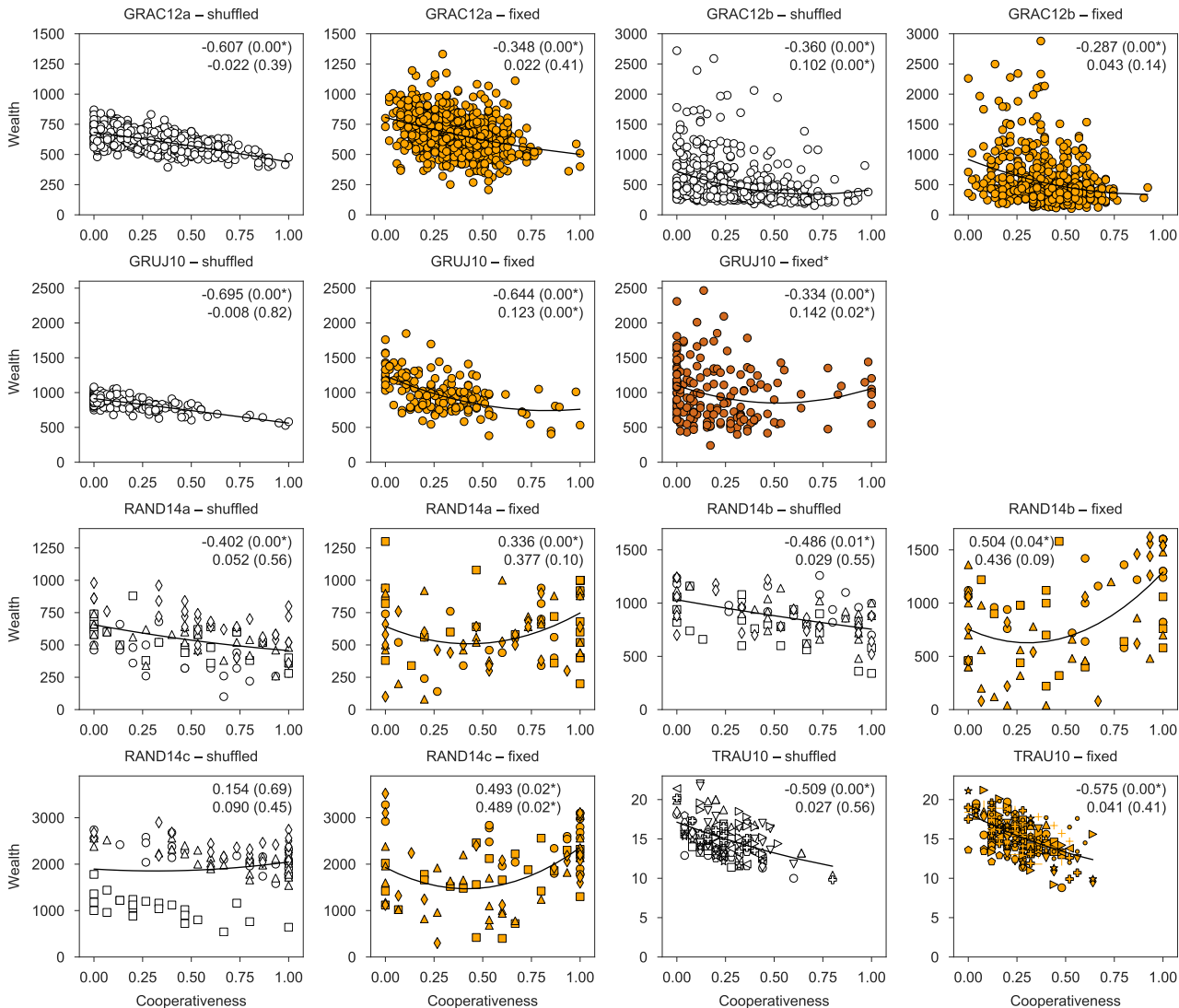

Supplement: S4 Fig — In each plot, values for individuals in the same interaction group are shown with the same symbol. The figure also shows fitted curves and estimates from ordinary least-square regressions (standardized regression coefficient for linear term on top and quadratic term on bottom, including p-values in brackets, with asterisk if p < 0.05). The standard errors in the regression models are estimated with correction for clustering by experimental group. (PDF) [file pone.0200965.s004.pdf]

CASA09

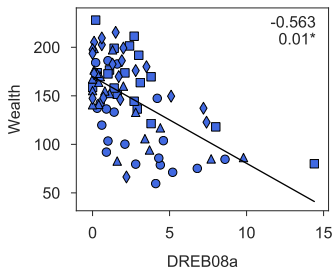

CASA09 – sequential

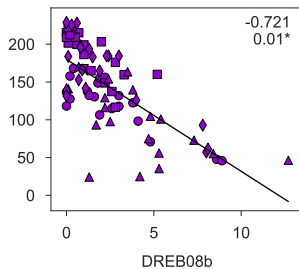

CASA09 – consensual

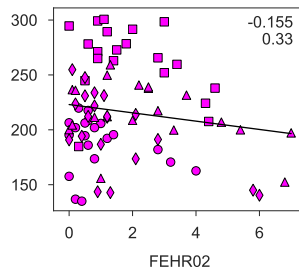

DREB08a

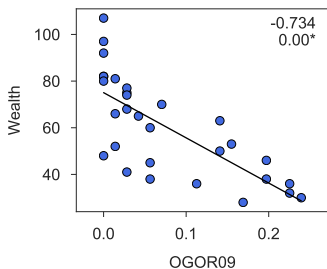

DREB08b

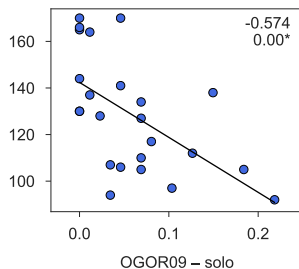

FEHR02

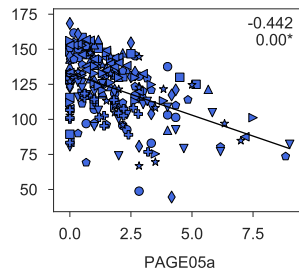

NIKI08

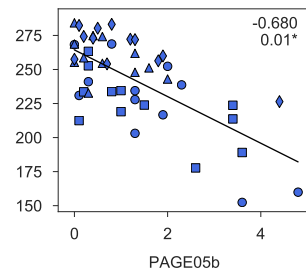

OGOR09

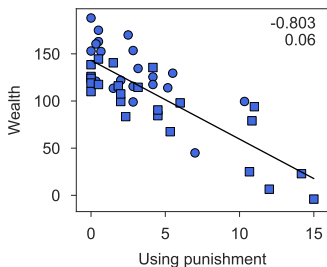

OGOR09 – solo

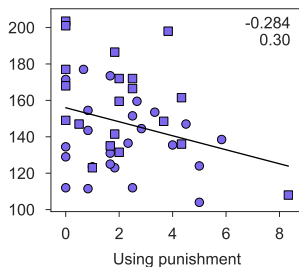

PAGE05a

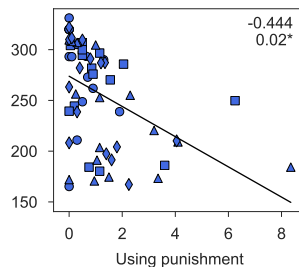

PAGE05b

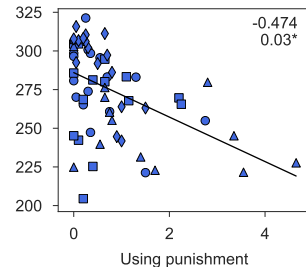

Supplement: S6 Fig — In each plot, values for individuals in the same interaction group are shown with the same symbol. The figure also shows fitted lines and estimates from ordinary least-square regressions (standardized regression coefficient, equivalent to the Pearson correlation, on top and p-value on bottom, with asterisk if p < 0.05). The standard errors in the regression models are estimated with correction for clustering by experimental group. (PDF) [file pone.0200965.s006.pdf]

CASA09

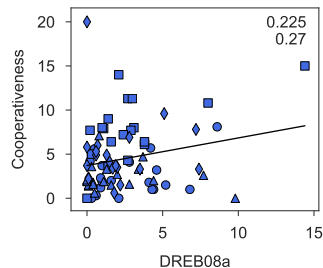

CASA09 – sequential

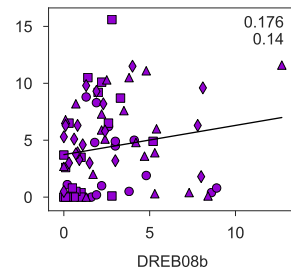

CASA09 – consensual

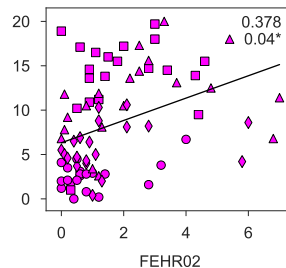

DREB08a

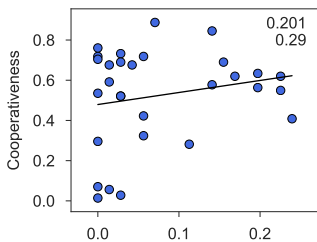

DREB08b

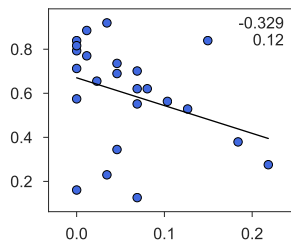

FEHR02

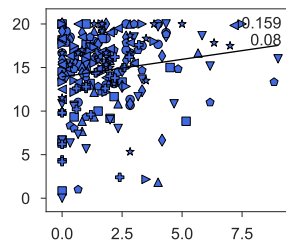

NIKI08

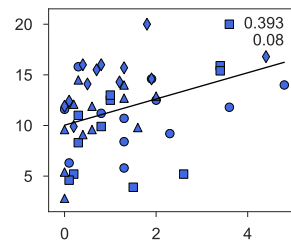

OGOR09

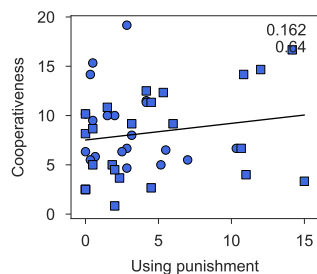

OGOR09 – solo

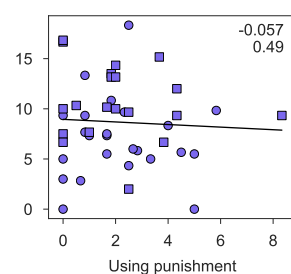

PAGE05a

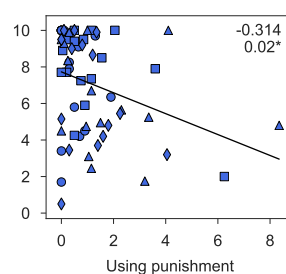

PAGE05b

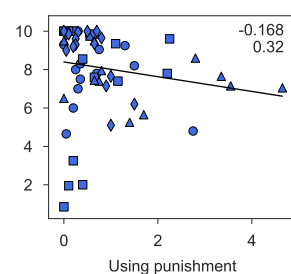

Supplement: S7 Fig — In each plot, values for individuals in the same interaction group are shown with the same symbol. The figure also shows fitted lines and estimates from ordinary least-square regressions (standardized regression coefficient, equivalent to the Pearson correlation, on top and p-value on bottom, with asterisk if p < 0.05). The standard errors in the regression models are estimated with correction for clustering by experimental group. (PDF) [file pone.0200965.s007.pdf]

CASS07

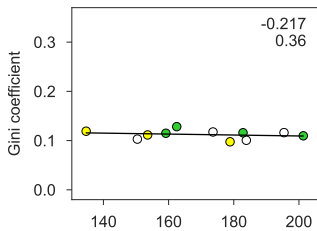

KIRC07a

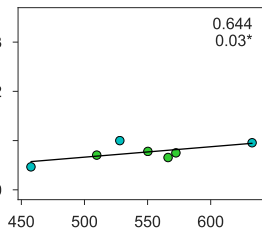

KIRC07b

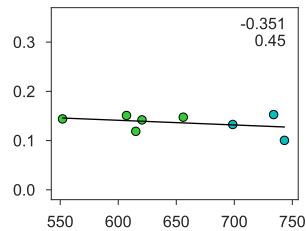

SURI11

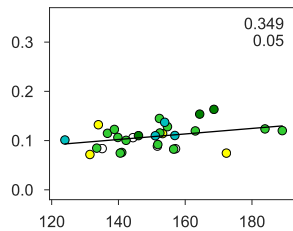

WANG12

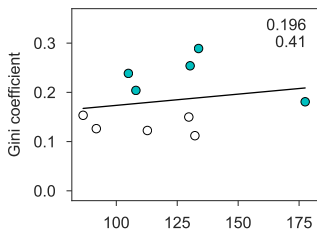

GRAC12a

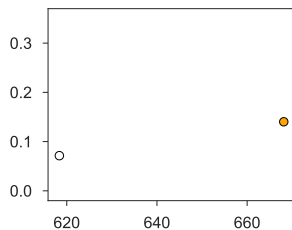

GRAC12b

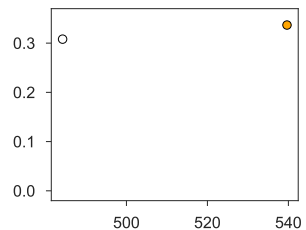

GRUJ10

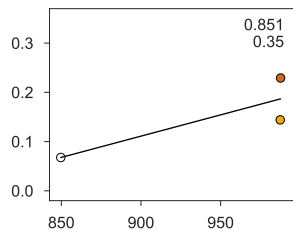

RAND14a

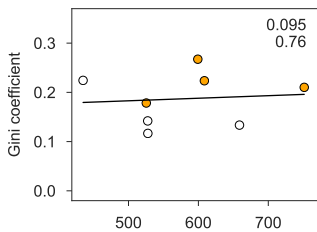

RAND14b

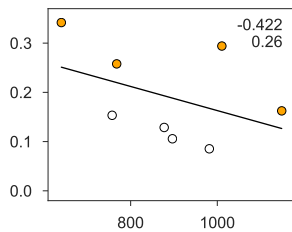

RAND14c

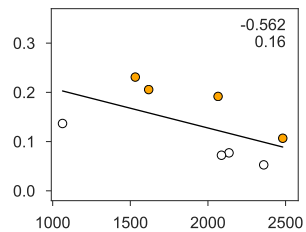

TRAU10

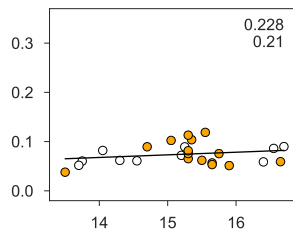

PAGE05a

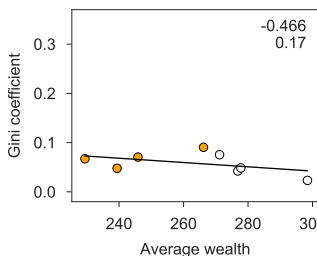

PAGE05b

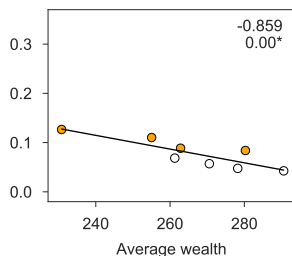

WANG12a

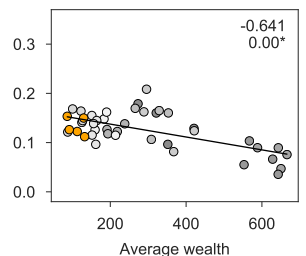

WANG12b

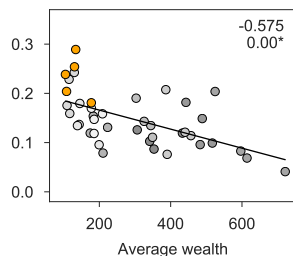

Supplement: S8 Fig — The figure shows fitted lines and estimates from ordinary least-square regressions (standardized regression coefficient, equivalent to the Pearson correlation, on top and p-value on bottom, with asterisk if p < 0.05). The standard errors in the regression models are estimated with correction for clustering by experimental group. Colors correspond to experimental conditions as in Fig 1A–1C. (PDF) [file pone.0200965.s008.pdf]

BOLT05a

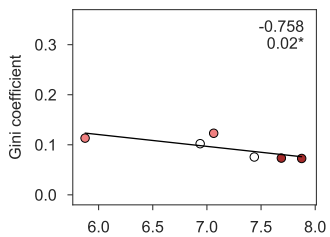

BOLT05b

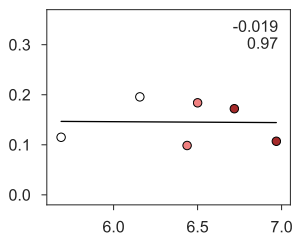

CUES15

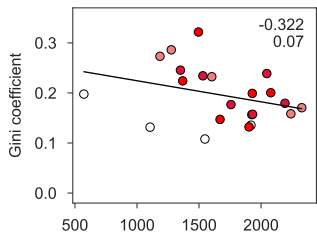

KAME17a

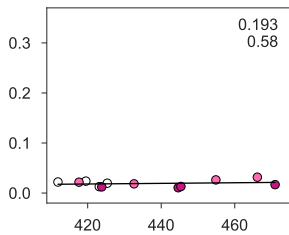

KAME17b

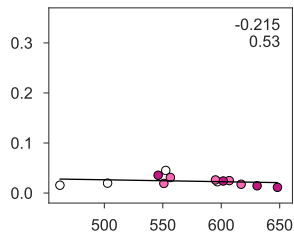

KIRC07a

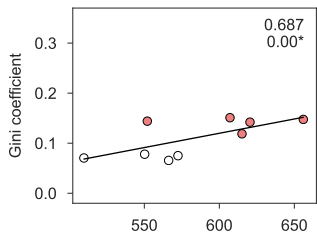

KIRC07b

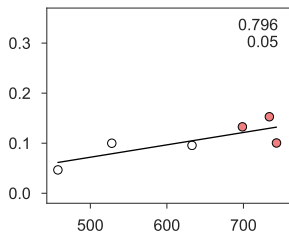

KIRC07c

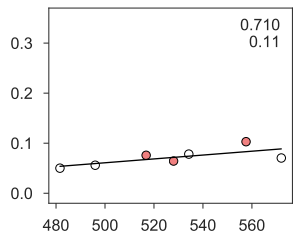

SEIN06

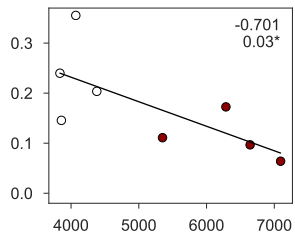

CASA09

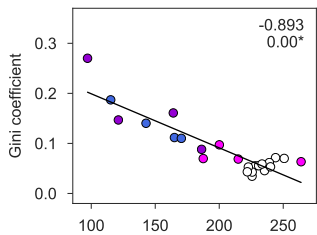

DREB08a

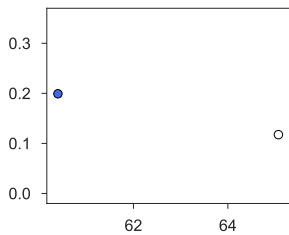

DREB08b

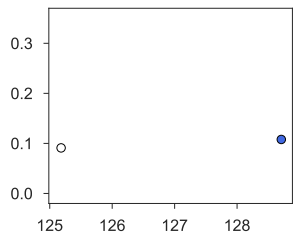

FEHR02

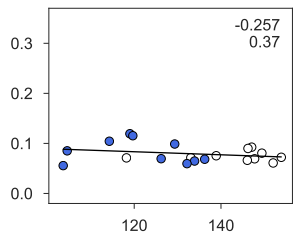

NIKI08

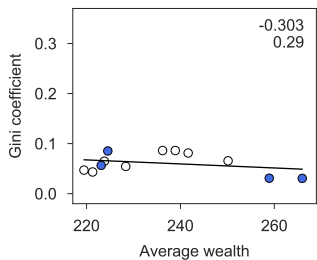

OGOR09

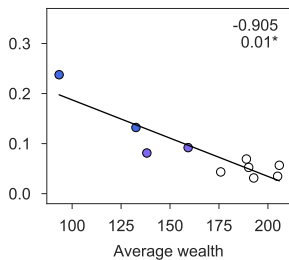

PAGE05a

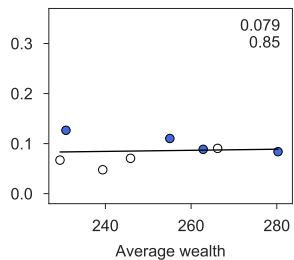

PAGE05b

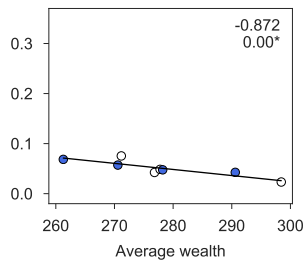

Supplement: S9 Fig — The figure shows fitted lines and estimates from ordinary least-square regressions (standardized regression coefficient, equivalent to the Pearson correlation, on top and p-value on bottom, with asterisk if p < 0.05). The standard errors in the regression models are estimated with correction for clustering by experimental group. Colors correspond to experimental conditions as in Fig 1D and 1E. (PDF) [file pone.0200965.s009.pdf]
