# Supplementary material for: The emergence of inequality in social groups: Network structure and institutions affect the distribution of earnings in cooperation games
Source: PLoS One. 2018 Jul 20;13(7):e0200965. doi: 10.1371/journal.pone.0200965 (PMC6054378; doi:10.1371/journal.pone.0200965)
Supplement: S3 Fig — In each plot, values for individuals in the same interaction group are shown with the same symbol. The figure also shows fitted curves and estimates from ordinary least-square regressions (standardized regression coefficient for linear term on top and quadratic term on bottom, including p-values in brackets, with asterisk if p < 0.05). The standard errors in the regression models are estimated with correction for clustering by experimental group. (PDF) [file pone.0200965.s003.pdf]

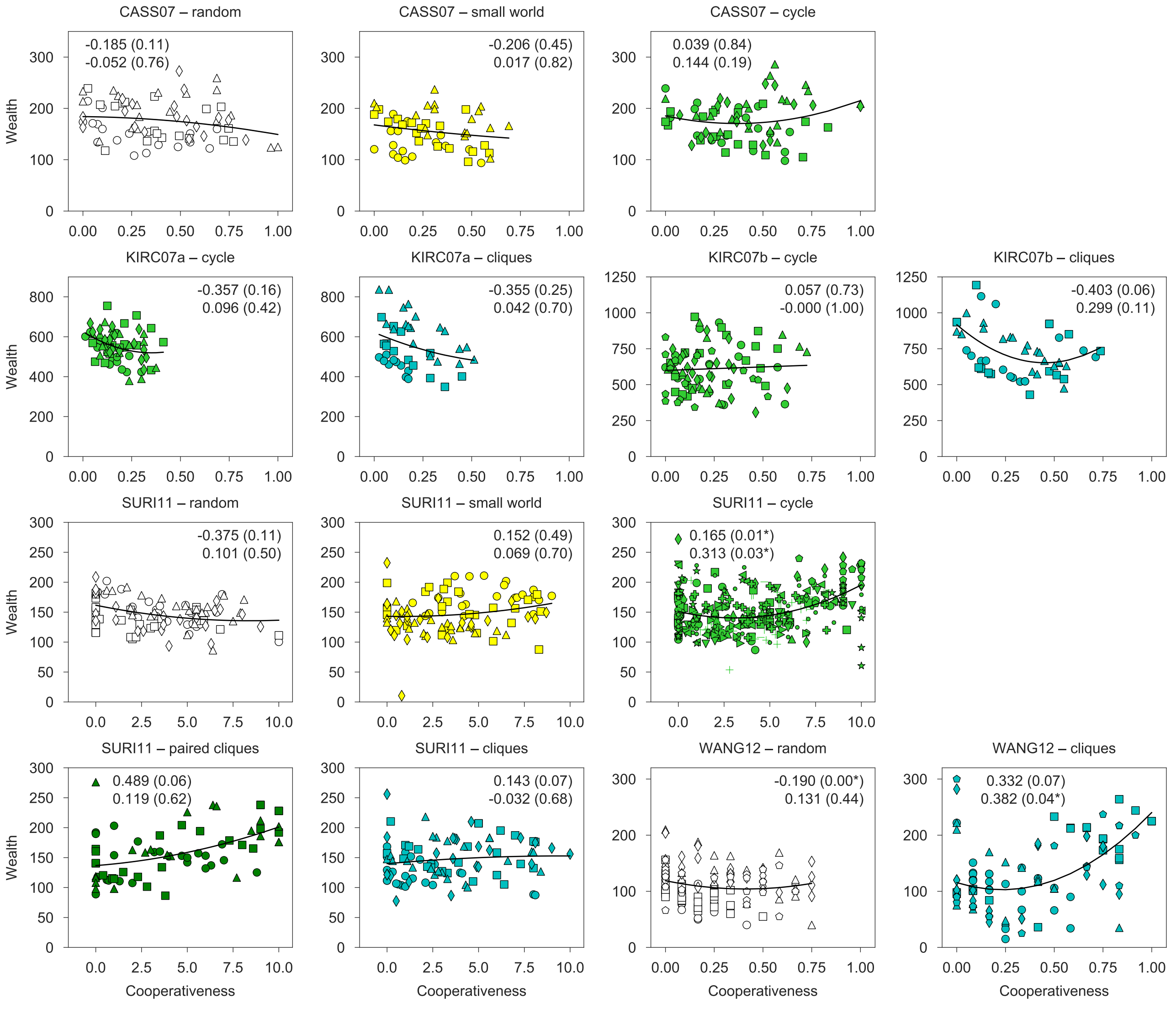

CASS07 – random
-0.185 (0.11)
-0.052 (0.76)
CASS07 – small world
-0.206 (0.45)
0.017 (0.82)
CASS07 – cycle
0.039 (0.84)
0.144 (0.19)
KIRC07a – cycle
-0.357 (0.16)
0.096 (0.42)
KIRC07a – cliques
-0.355 (0.25)
0.042 (0.70)
KIRC07b – cycle
0.057 (0.73)
-0.000 (1.00)
KIRC07b – cliques
-0.403 (0.06)
0.299 (0.11)
SURI11 – random
-0.375 (0.11)
0.101 (0.50)
SURI11 – small world
0.152 (0.49)
0.069 (0.70)
SURI11 – cycle
0.165 (0.01*)
0.313 (0.03*)
SURI11 – paired cliques
0.489 (0.06)
0.119 (0.62)
SURI11 – cliques
0.143 (0.07)
-0.032 (0.68)
WANG12 – random
-0.190 (0.00*)
0.131 (0.44)
WANG12 – cliques
0.332 (0.07)
0.382 (0.04*)
Wealth
Cooperativeness
