# Supplementary material for: The emergence of inequality in social groups: Network structure and institutions affect the distribution of earnings in cooperation games
Source: PLoS One. 2018 Jul 20;13(7):e0200965. doi: 10.1371/journal.pone.0200965 (PMC6054378; doi:10.1371/journal.pone.0200965)
Supplement: S5 Fig — In each plot, values for individuals in the same interaction group are shown with the same symbol. The figure also shows fitted curves and estimates from ordinary least-square regressions (standardized regression coefficient for linear term on top and quadratic term on bottom, including p-values in brackets, with asterisk if p < 0.05). The standard errors in the regression models are estimated with correction for clustering by experimental group. (PDF) [file pone.0200965.s005.pdf]

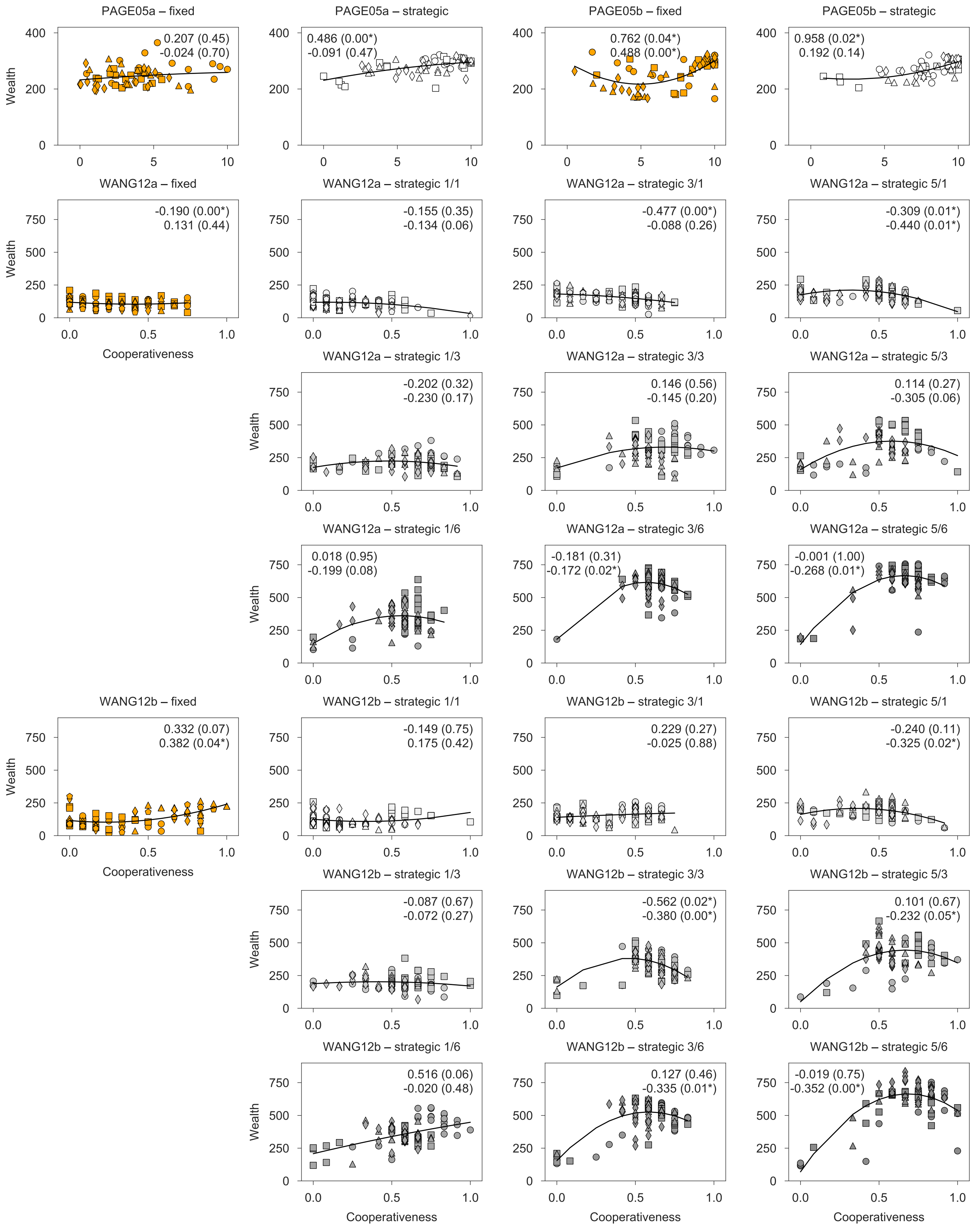

PAGE05a – fixed
0.207 (0.45)
-0.024 (0.70)
PAGE05a – strategic
0.486 (0.00*)
-0.091 (0.47)
PAGE05b – fixed
0.762 (0.04*)
0.488 (0.00*)
PAGE05b – strategic
0.958 (0.02*)
0.192 (0.14)
WANG12a – fixed
-0.190 (0.00*)
0.131 (0.44)
WANG12a – strategic 1/1
-0.155 (0.35)
-0.134 (0.06)
WANG12a – strategic 3/1
-0.477 (0.00*)
-0.088 (0.26)
WANG12a – strategic 5/1
-0.309 (0.01*)
-0.440 (0.01*)
WANG12a – strategic 1/3
-0.202 (0.32)
-0.230 (0.17)
WANG12a – strategic 3/3
0.146 (0.56)
-0.145 (0.20)
WANG12a – strategic 5/3
0.114 (0.27)
-0.305 (0.06)
WANG12a – strategic 1/6
0.018 (0.95)
-0.199 (0.08)
WANG12a – strategic 3/6
-0.181 (0.31)
-0.172 (0.02*)
WANG12a – strategic 5/6
-0.001 (1.00)
-0.268 (0.01*)
WANG12b – fixed
0.332 (0.07)
0.382 (0.04*)
WANG12b – strategic 1/1
-0.149 (0.75)
0.175 (0.42)
WANG12b – strategic 3/1
0.229 (0.27)
-0.025 (0.88)
WANG12b – strategic 5/1
-0.240 (0.11)
-0.325 (0.02*)
WANG12b – strategic 1/3
-0.087 (0.67)
-0.072 (0.27)
WANG12b – strategic 3/3
-0.562 (0.02*)
-0.380 (0.00*)
WANG12b – strategic 5/3
0.101 (0.67)
-0.232 (0.05*)
WANG12b – strategic 1/6
0.516 (0.06)
-0.020 (0.48)
WANG12b – strategic 3/6
0.127 (0.46)
-0.335 (0.01*)
WANG12b – strategic 5/6
-0.019 (0.75)
-0.352 (0.00*)
Wealth
Cooperativeness
